# Relevant Applications of Generative Adversarial Networks in Drug Design and Discovery: Molecular *De Novo* Design, Dimensionality Reduction, and *De Novo* Peptide and Protein Design

**DOI:** 10.3390/molecules25143250

**Published:** 2020-07-16

**Authors:** Eugene Lin, Chieh-Hsin Lin, Hsien-Yuan Lane

**Affiliations:** 1 Department of Biostatistics, University of Washington, Seattle, WA 98195, USA; lines@uw.edu; 2 Department of Electrical & Computer Engineering, University of Washington, Seattle, WA 98195, USA; 3 Graduate Institute of Biomedical Sciences, China Medical University, Taichung 40402, Taiwan; 4 Department of Psychiatry, Kaohsiung Chang Gung Memorial Hospital, Chang Gung University College of Medicine, Kaohsiung 83301, Taiwan; 5 School of Medicine, Chang Gung University, Taoyuan 33302, Taiwan; 6 Department of Psychiatry, China Medical University Hospital, Taichung 40447, Taiwan; 7 Brain Disease Research Center, China Medical University Hospital, Taichung 40447, Taiwan; 8 Department of Psychology, College of Medical and Health Sciences, Asia University, Taichung 41354, Taiwan

**Keywords:** artificial intelligence, deep learning, *de novo* peptide and protein design, dimension reduction, drug design, generative adversarial networks, machine learning, molecular *de novo* design, single-cell RNA sequencing

## Abstract

A growing body of evidence now suggests that artificial intelligence and machine learning techniques can serve as an indispensable foundation for the process of drug design and discovery. In light of latest advancements in computing technologies, deep learning algorithms are being created during the development of clinically useful drugs for treatment of a number of diseases. In this review, we focus on the latest developments for three particular arenas in drug design and discovery research using deep learning approaches, such as generative adversarial network (GAN) frameworks. Firstly, we review drug design and discovery studies that leverage various GAN techniques to assess one main application such as molecular *de novo* design in drug design and discovery. In addition, we describe various GAN models to fulfill the dimension reduction task of single-cell data in the preclinical stage of the drug development pipeline. Furthermore, we depict several studies in *de novo* peptide and protein design using GAN frameworks. Moreover, we outline the limitations in regard to the previous drug design and discovery studies using GAN models. Finally, we present a discussion of directions and challenges for future research.

## 1. Introduction

Nowadays researchers have been making compelling progress in the interdisciplinary fields of artificial intelligence, machine learning, and drug design and discovery [1,2,3,4]. In the arena of drug design and discovery, the goal of artificial intelligence and machine learning approaches is to provide data-driven algorithms that can in general help facilitate various stages of the drug development pipeline, such as drug target prediction, drug screening and discovery, preclinical trials, and clinical trials [2,5,6]. Latest advancements in artificial intelligence and machine learning technologies, especially deep learning algorithms [7,8], have exposed their encouraging quantities with respect to drug design and discovery [1,2,3,4,5,6,7,8,9,10,11,12,13]. For instance, in the preclinical stage of the drug development pipeline, deep learning approaches such as deep variational autoencoder [14] have been used to conduct the dimension reduction task of single-cell data for cell-specific biomarker discovery with single-cell RNA sequencing (scRNA-seq) techniques [15,16]. Furthermore, another interesting example of deep learning approaches is the generation of novel chemical structures by using deep variational autoencoder during the drug screening and discovery stage [17]. Thus, it has been suggested that deep learning approaches play a pivotal role in the future of drug design and discovery because their relevant applications encompass many aspects of drug design and discovery [3,18].

Principally, deep learning approaches incorporate the advanced artificial intelligence and machine learning models which utilize numerous layers of abstraction to build up hierarchical portrayals for the data [19,20,21]. For example, artificial neural networks can be utilized to establish the hierarchical representation [21,22]. In other words, deep learning approaches are comprised of computer programs that resolve the best predictions by using artificial neural networks with numerous layers, instead of applying artificial neural networks with only one individual layer [21]. Based on the state-of-the-art computing technologies (for example, general-purpose computing on graphics processing units), deep learning approaches have achieved a wide range of applications in drug design and discovery [9,20]. In order to address the demanding challenges we face today in the field of drug design and discovery, there is an enormous need for employing software tools in deep learning frameworks for various drug development tasks [2]. Namely, deep learning frameworks are employed to serve as tools to fulfill the applications of drug design and discovery, such as molecular *de novo* design, dimension reduction of single-cell data in preclinical development, compound property and activity prediction, reaction analysis, synthesis prediction, and biological image analysis [1].

With the recent advance in deep learning frameworks, the generative adversarial network (GAN) architecture [23] is an emerging technique that has attracted increasing attention in artificial intelligence and machine learning research. First, the GAN architecture possesses a tremendous potential to be leveraged in numerous applications, such as drug design and discovery, images, videos, languages, and other fields [24,25,26,27]. Moreover, the application of the GAN architecture has been contributing to drug design and discovery research. In the recent past, there have been a wide variety of vital research studies for drug design and discovery, such as molecular *de novo* design, with consideration of the GAN architecture [2,9]. For example, the GAN-based frameworks such as the deep adversarial autoencoder structure have been utilized to develop and identify novel compounds for anticancer therapy with chemical and biological datasets [28,29]. In addition, another remarkably intriguing example is that the deep adversarial variational autoencoder structure has shown to fulfill the task of dimensionality reduction for single-cell RNA sequencing data in the preclinical stage of the drug development pipeline [27]. In the following sections, we elaborate the details of various GAN-based frameworks such as the deep adversarial autoencoder and deep adversarial variational autoencoder structures in drug design and discovery.

Here, in the context of the GAN-based frameworks, we provide various research studies with focus on three major categories in terms of drug design and discovery including molecular *de novo* design, dimension reduction of single-cell data in preclinical development, and *de novo* peptide and protein design. We mainly focus on these three applications using a wide variety of the GAN-based frameworks because, to our knowledge, there may be scant studies in drug design and discovery using the GAN-based frameworks for other applications at the time of the submission of this paper. Accordingly, biological and/or clinical implications from these three major arenas could then serve as a basis for future research in drug design and discovery using the GAN-based frameworks. Additionally, we present the limitations in these research studies and summarize a discussion of future challenges as well as directions. While this review does not support the full set of related research studies reported in the literature, it nonetheless describes a synthesis of those that can markedly influence public and population health-oriented applications in drug design and discovery using the GAN-based frameworks in the near to mid-term future.

## 2. Generative Adversarial Network (GAN) Architecture

At first, Goodfellow et al. [23] introduced the concept of the GAN architecture as a form of generative models in an adversarial way. Since then, the GAN architecture has turned into one of interesting and hot topics in the field of artificial intelligence and machine learning [30,31]. Especially, the GAN architecture has become state-of-the-art in the field of computer vision and image processing (such as image generation) where astonishing progresses have been achieved [30,31]. Because there is a flood of publications on numerous variants of the GAN architecture in different branches of science and engineering, it is really challenging to follow the emerging trend [30,31].

The GAN architecture offers the following advantages. First, based on the empirical experiments, the GAN architecture often provides better results than other generative methods [30]. Second, the GAN architecture can conduct the sampling task in parallel, which contributes a substantial speedup for producing samples [30]. Third, real data distributions or mathematical conditions are not required to perform the GAN architecture [23].

### 2.1. Brief Description of the GAN Architecture

**Figure 1:**
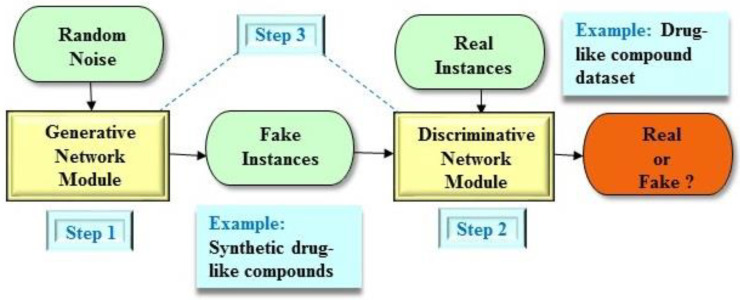
An example of the generative adversarial network (GAN) architecture. The GAN architecture comprises two main components including a generative network module and a discriminative network module. Step 1: The generative network module produces synthetic instances as real as possible. Gaussian random noises normally serve as the input for the generative network module. One particular example in drug design and discovery is a reconstructed drug-like compound as a fake instance. Step 2: The discriminative network module assesses the probability that an instance stems from the real dataset. One particular example in drug design and discovery is a drug-like compound dataset. Step 3: Both the generative and discriminative network modules play concurrently against each other to obtain their objectives.

In brief, the GAN architecture consists of two fundamental elements including a generative network module and a discriminative network module (Figure 1) [23]. Basically, these generative and discriminative network modules are two artificial neural networks with multiple layers, which are trained concurrently. While the generative network module is trained to produce fake instances based on the latent variable, the discriminative network module receives both real and fake instances and differentiates whether its input is real or not. The discriminative network module predicts higher probability if it recognizes that an instance is more inclined to be real. At the same time, the generative network module is trained to boost the probability of the discriminative network module making a mistake. That is, both the generative and discriminative networks play simultaneously against each other to realize their goals. Consequently, the GAN architecture achieves an adversarial game between the generative and discriminative network modules. This scheme can be formalized as the following type of minimax objective [23]:

(1) $$\underset{G}{\min}\;\underset{D}{\max}\;E_{x\sim P_{data}}\left[\log D\left(x\right)\right]+E_{z\sim P\left(z\right)}\left[\log\left(1-D\left(G\left(z\right)\right)\right)\right]$$

where *G* is the generative network module, *D* is the discriminative network module, *p_data_* is the data distribution, *x* is a point in the data space, *z* represents samples, and *p*(*z*) is the model distribution.

### 2.2. Applications of the GAN Architecture

There are a wide range of applications by using the GAN architecture, including computer vision, image processing, medical imaging, music, speech processing, and video processing, to name a few [30,31]. In addition, the GAN architecture has been applied in chemistry (such as chemoinformatics and molecular informatics) and biology (such as drug design and discovery, bioinformatics, medical informatics, and multi-omics) [30,31].

### 2.3. Variants of the GAN Architecture

There are a wide variety of the GAN-based frameworks (that is, variants of the GAN architecture). In this section, we introduce the following three variants: the Wasserstein GAN structure, the conditional GAN structure, and the deep adversarial autoencoder structure. In addition, the reader can refer to recent reviews by Alqahtani et al. [30] and Lan et al. [31] for other variants of the GAN architecture.

#### 2.3.1. Wasserstein GAN

To overcome the instability of GAN training, Arjovsky et al. [32] proposed the Wasserstein GAN structure, which utilizes a new distance measurement method called the Earth-Mover distance (also known as Wasserstein distance). On the other hand, the original GAN architecture employs the Jensen–Shannon divergence which, in theory, is inapplicable to estimate the distance between two distributions if the distributions do not overlap [32].

#### 2.3.2. Conditional GAN

Mirza et al. [33] proposed the conditional GAN structure, where both the generative and discriminative network modules are conditioned on some auxiliary information such as class labels. Unlike the original GAN architecture, the conditional GAN structure is a supervised method by using a conditional variable (for example, class labels). The conditional variable serves as an additional input for both the generative and discriminative network modules. While the generative network module is trained to produce fake instances based on the latent variable and class labels, the discriminative network module learns the correlation between class labels and instances.

#### 2.3.3. Adversarial Autoencoder

**Figure 2:**
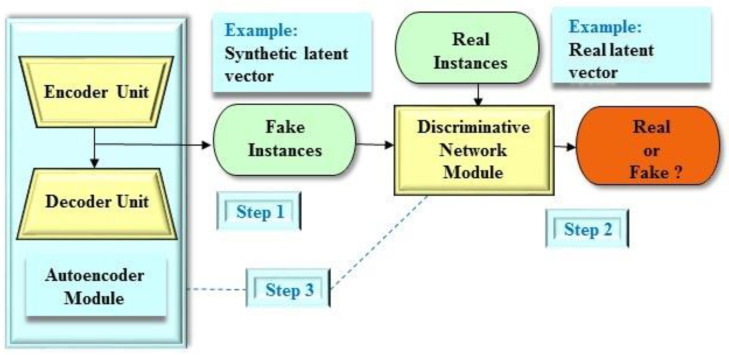
An example of the deep adversarial autoencoder structure. The deep adversarial autoencoder structure comprises two main components including an autoencoder module and an adversarial network module. The autoencoder module comprises an encoder unit and a decoder unit. The encoder unit also serves as the generative network module of the adversarial network architecture. Step 1: The encoder unit produces synthetic instances as real as possible. One particular example in drug design and discovery is a reconstructed latent vector as a fake instance. Step 2: The discriminative network module assesses the probability that an instance stems from the real dataset. One particular example in drug design and discovery is a real latent vector from the drug-like compound dataset. Step 3: Both the autoencoder and discriminative network modules play concurrently against each other to obtain their objectives.

From a structural point of view, the deep adversarial autoencoder structure [34], which is a variant of the GAN-based frameworks, is a probabilistic autoencoder structure that employs the GAN architecture to transform an autoencoder structure into a generative model. Essentially, the deep adversarial autoencoder structure is comprised of two components, a conventional autoencoder module and an adversarial network module (Figure 2). Moreover, the autoencoder module consists of two components, an encoder unit and a decoder unit. In the deep adversarial autoencoder structure, the encoder unit is also considered as the generative network module of the adversarial network architecture. Furthermore, the concept of the deep adversarial autoencoder structure is that both the adversarial network and autoencoder modules are trained concurrently to complete the tasks. In addition, the generative network module (that is, the encoder unit) is constructed to set a trap that will lead the discriminative network module to believe the information (that is, the latent vector) generated by the generative network module. On the contrary, the discriminative network module is designed to differentiate between the ground true data and the information (that is, the latent vector) of the generative network module (that is, the encoder unit), simultaneously. On the whole, the objective of the training in the deep adversarial autoencoder structure is to make sure that the latent data generated by the generative network module matches with the specific prior latent distribution.

On the other hand, the variational autoencoder structure [14,35], which is a variant of the autoencoder-based frameworks, is a generative model for estimating the probability density function of the training data. Basically, the variational autoencoder structure consists of two components, an encoder unit and a decoder unit. It should be noted that the variational autoencoder structure does not have an adversarial network module. The objective of the encoder unit is to implement the mean and covariance of the Gaussian distribution to serve as the variational distribution in the variational autoencoder structure [14]. A recent study by Lin et al. [27] incorporated the variational autoencoder structure with the deep adversarial autoencoder structure to create the deep adversarial variational autoencoder structure, which is described in Section 4 in this review.

## 3. Molecular *De Novo* Design

Molecular *de novo* design has a long history dating back to about 25 years ago with an aim to create novel active molecules during the drug screening and discovery stage in the drug development pipeline [3]. The usage of the GAN architecture is still in its infancy in terms of molecular *de novo* design. Recently, GAN-based applications in molecular *de novo* design have experienced some revival in the field of artificial intelligence and machine learning. Here, we focus on molecular *de novo* design using various GAN-based strategies in this section (Table 1). In this review, we first conducted a comprehensive search of the electronic PubMed database (2015–present) using key words such as “generative adversarial network,” “deep learning,” “drug design and discovery,” and “molecular *de novo* design”. Then, we manually screened the obtained articles with a particular focus on the GAN architecture and molecular *de novo* design.

In addition, the reader can refer to a recent review by Chen et al. [1] for studies that apply the recurrent neural network model [46] and the reinforcement learning technique to molecular *de novo* design, where the recurrent neural network model [46] and the reinforcement learning technique are also defined as deep learning approaches, but not the focus of this review. The reader can also refer to a recent review by Hessler and Baringhaus [3] for other aspects of deep learning models in molecular *de novo* design, such as the variational autoencoder structure [14] and the recurrent neural network model [46], which again are not the focus of this review. It is worthwhile to mention that deep reinforcement learning technique is sometime incorporated with the GAN architecture in several studies as mentioned in this section.

In molecular *de novo* design, molecular representation schemes are in the core of chemoinformatics because further downstream analysis depends on them [47]. Moreover, deep learning models using molecular representation schemes may benefit from the usage of high information content contained in the molecular representation schemes [47]. While many molecular representation schemes exist, the most popular molecular representation scheme is a string-based representation called simplified molecular-input line-entry system (SMILES) strings for representing a set of molecular compounds [48]. Other molecular representation schemes include image-based representations [49,50], graph-based representations [51], and tensor-based representations [52]. Briefly, the image-based representations are based on custom-generated 2D images to represent molecular features [49,50]. Moreover, molecular features are represented as multidimensional arrays (that is, tensors) in the tensor-based representations [52]. Furthermore, deep learning models can perform tasks using molecular graphs directly in the graph-based representations [51]. Another example of graph-based molecular representations is 166 bit MACCS (Molecular ACCess System) chemical keys (or fingerprints) [53], where each bit is linked with a distinct structural arrangement about a molecular structure.

**Figure 3:**
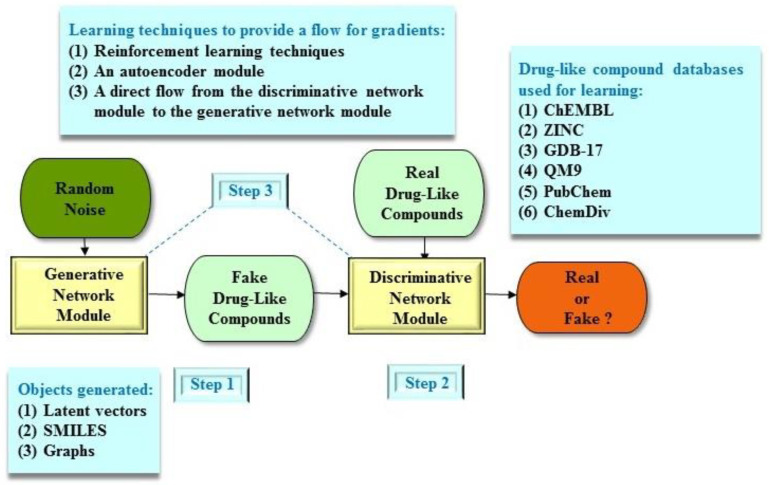
An example of a workflow of the generative adversarial network (GAN) architecture for molecular *de novo* design. Step 1: The generative network module produces synthetic drug-like compounds (which are generated as latent vectors, SMILES, or graphs) as real as possible. Step 2: The discriminative network module assesses the probability that a drug-like compound stems from the real drug-like compound datasets (for example, ChEMBL). Step 3: Both the generative and discriminative network modules play concurrently against each other to obtain their objectives. Note that the solutions to provide a flow for gradients include reinforcement learning techniques, an autoencoder module, and a direct flow.

The GAN architecture is able to outperform other architectures only when the generative network module can produce continuous output values (such as a vector of numbers as in image generation). In this case, we are able to train the generative network module and adjust its weights by using the gradient of the loss function from the discriminative network module. However, chemical structures (that is, molecular compounds) could not be represented in continuous numbers and are represented using text strings (such as SMILES) or molecular graphs. This is one of the most important complications of GAN-based applications in chemistry (or chemoinformatics). Therefore, we have to invent a way to facilitate gradients through chemical structures, which are represented as SMILES or graphs (Figure 3). The existing solutions in the literature are as follows. First, a reinforcement learning approach is employed to provide policy gradients for adjusting weights in the generative network module, which in turn generates chemical structures. Second, an autoencoder module (including an encoder unit and a decoder unit) is utilized to serve as a translator, which encodes chemical structures into latent vectors and then decodes latent vectors back to chemical structures. Third, chemical structures are not generated explicitly. Both the generative and discriminative network modules work directly with latent vectors, which then can be translated back to chemical structures. Finally, the adversarial autoencoder structure is utilized because it does not have the generative network module, but it has an autoencoder module.

In addition, a bioactive molecular database called the ChEMBL database (https://www.ebi.ac.uk/chembl/) is often utilized to facilitate the molecular *de novo* design process, where the ChEMBL database is a manually curated database maintained by the European Bioinformatics Institute to help effectively design new drugs. Other public databases for commercially available compounds and combinatorially generated libraries include the ZINC (http://zinc15.docking.org) [54], GDB-17 (http://gdb.unibe.ch/downloads) [55], QM9 (http://quantummachine.org/datasets/) [56], PubChem (https://pubchem.ncbi.nlm.nih.gov) [57], and ChemDiv (http://www.chemdiv.com/) databases.

To generate new molecules with desired molecular features, Kadurin et al. [28,29] investigated the druGAN (drug Generative Adversarial Network) structure by leveraging the deep adversarial autoencoder structure [34], which represents a variant of the GAN-based frameworks. And the PubChem drug-like compound database [57] was utilized. Firstly, the druGAN structure is comprised of fully connected deep neural network algorithms. Furthermore, Kadurin et al. [28,29] carried out experiments based on 166-bit MACCS chemical fingerprints [53] to extract molecular features with specific properties. In addition, the druGAN (or deep adversarial autoencoder) structure, which contains an autoencoder module, was able to provide a way to facilitate gradients through chemical structures (represented as 166-bit MACCS chemical fingerprints). The benefit of this deep learning GAN architecture is that the druGAN structure is able to generate new chemical compounds which can be considered as potential anticancer agents. Moreover, Kadurin et al. [28,29] revealed that the druGAN structure outperformed the variational autoencoder structure [14] in terms of the capacity and efficiency of the models. It should be noted that the variational autoencoder structure is also a deep learning approach, but not a GAN framework.

To generate and identify new chemical compounds, various research studies have incorporated the GAN methods with the reinforcement learning technique [58]. The idea of integrating the neural network structures with the reinforcement learning technique dates back to 30 years ago [59]. Recently, with the advances in deep neural network models, the reinforcement learning technique is emerging again. Previously, the reinforcement learning technique can only be successfully applied to the problems with the low-dimensional spaces. With the help of deep neural network models, the so-called deep reinforcement learning technique is able to handle useful applications in the high-dimensional spaces [60]. In general, the idea of the reinforcement learning technique can be implemented by applying the recurrent neural network model [46]. In terms of structure, the recurrent neural network model is a variant of artificial neural networks, which displays sequentially-progressive transition by using a directed graph with a temporal sequence [46].

For example, Guimaraes et al. [36] suggested that the ORGAN (Objective-Reinforced Generative Adversarial Networks) structure, a combination method involving the GAN and reinforcement learning technologies, was able to generate novel molecular compounds with preferred properties in the context of drug development pipeline. It should be noted that the ORGAN structure employed a reinforcement learning method for yielding policy gradients to adjust the weights of the generative network module, where molecules were encoded by using the SMILES representation. The ORGAN structure utilized drug-like compound databases such as the ZINC [54] and GDB-17 [55] databases. In terms of architecture, the concept of the ORGAN structure is comprised of the Sequence GAN model [61] and the recurrent neural network model [46], where the Sequence GAN model is a variant of the GAN-based frameworks. In addition, the discriminative network module is implemented as the convolutional neural network model [62], and the generative network module is constructed as the recurrent neural network model [46]. Moreover, the recurrent neural network model consists of the repeating modules of neural networks that can perform the analysis of progressive changes through time [46]. In particular, the ORGAN structure displayed better results than the recurrent neural network model or the GAN model alone did.

Moreover, the subsequent study by Sanchez-Lengeling et al. [37] reported that the ORGANIC (Objective-Reinforced Generative Adversarial Network for Inverse-design Chemistry) structure, a revised version of the ORGAN structure, was able to represent molecular compounds as strings for inverse design of molecular compounds. In accordance with ORGAN, the ORGANIC structure also employed a reinforcement learning technique to generate policy gradients for adjusting the weights of the generative network module, where molecules were encoded by using the SMILES representation. Drug-like compound databases such as the ZINC [54] and GDB-17 [55] databases were utilized. In terms of architecture, the ORGANIC structure is similar to the ORGAN structure, where the discriminative network module is represented as the convolutional neural network model [62] and the generative network module is implemented as the recurrent neural network model [46]. Consequently, the ORGANIC structure demonstrated good performance on the quantitative estimate of drug-likeness and generated 207 drug-like molecule compounds when compared with the drugs approved by the US Food and Drug Administration (FDA) [37]. It is worthwhile to mention that drug-likeness is a key factor to be considered when we screen molecular compounds during the early stages of drug discovery and design [63]. Additionally, the objective of drug-likeness [37] is to evaluate drug-like and non-drug-like molecules in terms of molecular compound properties such as absorption, distribution, metabolism, and excretion.

On another note, Putin et al. [38] showed that the RANC (Reinforced Adversarial Neural Computer) structure, a combination method involving the GAN and reinforcement learning technologies, had better performance when compared with the ORGANIC structure. In line with ORGAN and ORGANIC, the RANC structure also utilized a reinforcement learning approach to facilitate gradients via chemical structures (represented as the SMILES representation). In addition, the ZINC [54] and ChemDiv (http://www.chemdiv.com/) drug-like compound databases were used. While the ORGANIC structure used a long short-term memory unit [64] in the recurrent neural network model [46], the RANC structure employed a differentiable neural computer architecture which has been shown to outperform the long short-term memory unit [65]. Particularly, Putin et al. [38] found that the RANC structure was superior to the ORGANIC structure in terms of several drug discovery metrics, including the number of unique structures (3 times higher), medicinal chemistry filters (1.7 times higher), Muegge criteria (2.2 times higher) [66], and quantitative estimate of drug-likeliness scores (1.5 times higher) [63]. Moreover, one strength of the RANC structure is that it was shown to be stable and consistent during training.

In another study, Putin et al. [39] demonstrated that the ATNC (Adversarial Threshold Neural Computer) structure, a combination method involving the GAN and reinforcement learning technologies, utilized a specific unit called adversarial threshold to overcome the negative reward problem of reinforcement learning in the ORGANIC structure. It should be mentioned that the RANC structure also utilized a reinforcement learning method to facilitate gradients via chemical structures (represented as the SMILES representation) as in ORGAN, ORGANIC, and ATNC. Drug-like compound databases such as ChemDiv (http://www.chemdiv.com/) database were used. To sum up, the adversarial threshold unit is an additional discriminative network module which performs reinforcement learning tasks to enforce a positive reward in the environment. The ATNC structure was able to create 72% of valid SMILES strings and 77% of unique SMILES strings. Moreover, the ATNC structure was able to produce a higher percentage of unique SMILES strings (that is, molecular compounds) than the ORGANIC structure.

Furthermore, a study by Polykovskiy et al. [40] implicated that the Entangled Conditional Adversarial AutoEncoder (ECAAE) structure can be applied to generate new molecular compounds, which have specific properties of synthesis and solubility to serve as initial drug targets in the drug discovery pipeline. In line with druGAN, the ECAAE structure also used the adversarial autoencoder structure to provide gradients via chemical structures (represented as the SMILES representation). The ZINC drug-like compound database [54] was utilized. Firstly, the ECAAE structure was based on the conditional adversarial autoencoder model which is a variant of adversarial autoencoder [34] with the conditional generation [33] (that is, an additional condition for the input of the generative network module). Secondly, in order to have stable and better results, the ECAAE structure employs the concept of combined disentanglement, which includes both the predictive and joint disentanglement. Moreover, Polykovskiy et al. [40] revealed that the ECAAE structure was able to generate a novel molecular compound, which was demonstrated, in an in vitro study, to possess high binding affinity and specificity with the Janus kinase 3 protein for diseases such as rheumatoid arthritis, psoriasis, and vitiligo.

In a similar way, Cao and Kipf [41] also carried out a deep learning GAN architecture called the MolGAN (Molecular GAN) structure, which suggests molecular synthesis using graph-structured data directly. It is worthwhile to mention that the MolGAN structure employed a reinforcement learning algorithm (or a policy gradient algorithm) to adjust the generative network module via chemical structures (represented as undirected graphs). Drug-like compound databases such as the GDB-17 [55] and QM9 [56] databases were utilized. Basically, the MolGAN structure is comprised of a generative network module, a discriminative network module, and a reward network module. Additionally, the reward network module is utilized to provide an optimized molecule generation via the reward function by using the reinforcement learning technique. Moreover, the MolGAN structure employs the annotated molecular graphs, which correspond to individual chemical compounds, to serve as the input for the MolGAN structure. By using the QM9 molecular database, the MolGAN structure was compared with the ORGAN structure [36] and the variational autoencoder-based methods such as the CharacterVAE [67], GrammarVAE [68], and GraphVAE [52] structures. It was indicated that the MolGAN structure outperformed the ORGAN structure and these variational autoencoder-based methods.

In line with MolGAN [41], Guarino et al. [42] proposed the DiPol-GAN (Differentiable Pooling GAN) structure, which also suggests molecular synthesis using graph-structured data (that is, undirected graphs) directly. While MolGAN employs the Wasserstein GAN structure, the DiPol-GAN structure utilizes the Relational-GCN (Relational Graph Convolutional Network) model [69] to implement the generative network module, which generates the graph objects. In accordance with MolGAN, the DiPol-GAN structure also used a reinforcement learning objective, where a policy gradient algorithm was utilized to adjust the generative network module. It was indicated that the DiPol-GAN structure had 1.3 times higher drug-likeliness scores than the MolGAN structure by using the QM9 drug-like compound database.

Moreover, a study by Prykhodko et al. [43] showed that the LatentGAN (Latent vector based Generative Adversarial Network) structure, which is also a deep learning GAN architecture, can be utilized to perform *de novo* molecular generation tasks. In terms of architecture, the LatentGAN structure is a heteroencoder structure, which consists of a generative network module and a discriminative network module. In addition, the heteroencoder structure (which is similar to an autoencoder module) is trained with various non-canonical (or non-unique) SMILES strings of the same molecular compound. Here, the autoencoder module (including an encoder unit and a decoder unit) serves as a translator to encode chemical structures (represented as the SMILES representation) into latent vectors and then decode latent vectors back to chemical structures. In other words, the encoder unit of the heteroencoder structure is trained by using SMILES strings from the ChEMBL database (https://www.ebi.ac.uk/chembl/) to transform chemical structures to latent vectors. Then, the generated latent vectors from the encoder unit are utilized as the true data input for the discriminative network module. While the generative network module is trained to produce fake instances based on the latent vectors, the discriminative network module receives both real and fake instances and differentiates whether its input is real or not. After the training for both the generative network module and discriminative network module is ready, the generative network module produces the sampled latent vector for the decoder unit of the heteroencoder structure. Then, based on the sampled latent vector from the generative network module, the decoder unit creates the SMILES strings of the novel molecular compound. By using a randomly selected ChEMBL subset, Prykhodko et al. [43] demonstrated that the LatentGAN structure was able to generate novel drug-like compounds.

Likewise, Maziarka et al. [44] implemented a deep learning GAN architecture called the Mol-CycleGAN structure to produce optimized molecular compounds where their molecular structures were highly similar to the original ones. It should be emphasized that both the generative and discriminative network modules in the Mol-CycleGAN structure directly performed with latent vectors, and then the latent vectors were translated back to chemical structures (represented as molecular graphs). Drug-like compound databases such as the ZINC [54] and ChEMBL (https://www.ebi.ac.uk/chembl/) databases were utilized. Essentially, the concept of the Mol-CycleGAN structure stemmed from the CycleGAN structure [70], which was originally applied to image-to-image translation in computer vision research. By using the ZINC molecular database, the Mol-CycleGAN structure was compared with the junction tree variational autoencoder structure [71] and the graph convolutional policy network structure [51]. It was implicated that the Mol-CycleGAN structure outperformed the junction tree variational autoencoder and graph convolutional policy network structures in terms of the mean improvement of the compound property for drug-like molecules.

Finally, a recent study by Méndez-Lucio et al. [45] proposed a deep learning GAN architecture called the conditioned GAN structure to design novel molecular compounds with a chosen biological activity such as a transcriptomic profile. In line with Mol-CycleGAN, both the generative and discriminative network modules in the conditioned GAN structure precisely operated with latent vectors, and then the latent vectors were transformed back to chemical structures (represented as 166-bit MACCS chemical fingerprints [53]). The L1000 drug-like compound database [72] was utilized. In terms of architecture, the concept of the conditioned GAN structure stems from the conditional GAN structure [33] and the Wasserstein GAN structure with gradient penalty [32,73]. Under the conditional GAN structure, the objective of its generative network module is to provide the synthetic information to fulfill the predefined condition [33]. On the other hand, the Wasserstein GAN structure with gradient penalty [32,73] is a variant of the GAN-based frameworks that utilizes a minimization function with the Earth-Mover distance (namely Wasserstein-1 distance) instead of the Jensen–Shannon divergence. The conditioned GAN structure is also based on the convolutional neural network model [62], which was originally applied to text mining in natural language processing research [74]. It was suggested that the conditioned GAN structure was able to achieve molecular compounds with a desired gene expression signature by using the L1000 database [72]. It is also revealed that the conditioned GAN structure has advantages over the classical similarity search [75] such as a similarity search using Euclidean distance.

## 4. Dimension Reduction of Single-Cell Data in Preclinical Development

In the preclinical stage of the drug development pipeline, single-cell RNA sequencing (scRNA-seq) is an emerging technology that can be used to evaluate the function of an individual cell at the single cell level [2,27,76]. Dimensionality reduction is a crucial step prior to downstream analysis of scRNA-seq data in the preclinical development stage. Its goal is to transform data points from high dimensions (up to 30 thousands) to low dimensions (2 or 3) so that the data become more practicable in the smaller scale [27,77]. Several works have applied the GAN-based frameworks for dimensionality reduction in scRNA-seq analysis [27,78]. In this review, we first conducted a comprehensive search of the electronic PubMed database (2015–present) using key words such as “generative adversarial network,” “deep learning,” “dimensionality reduction,” and “single-cell RNA sequencing”. Then, we manually screened the obtained articles with a particular focus on the GAN-based frameworks and dimensionality reduction.

In addition, the reader can refer to a recent review by Zheng and Wang [79] for studies that apply other deep learning models to dimensionality reduction in scRNA-seq analysis, including the conventional machine learning approaches such as principal component analysis (PCA) [80] and the variational autoencoder structure [14]. Again, these approaches are not the focus of this review.

**Figure 4:**
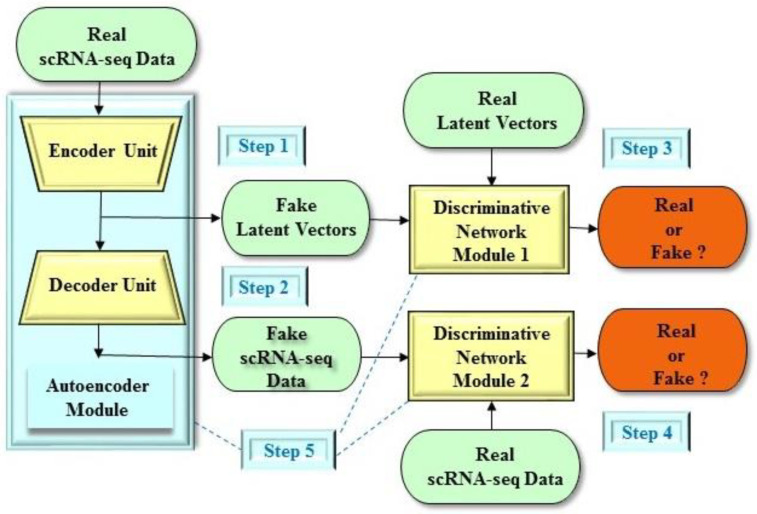
An example of the DR-A (Dimensionality Reduction with Adversarial variational autoencoder) model for dimensionality reduction in scRNA-seq analysis. Step 1: The encoder unit produces synthetic latent vectors as real as possible. The encoder unit provides the mean and covariance of the Gaussian distribution to serve as the variational distribution, which is commonly generated by a variational autoencoder structure. Step 2: On the other hand, the decoder unit produces reconstructed scRNA-seq data as real as possible. Step 3: The DR-A model has two discriminative network modules. The first discriminative network module assesses the probability that the latent vector stems from the real latent vectors. Step 4: The second discriminative network module assesses the probability that the scRNA-seq data stems from the real scRNA-seq datasets. (e) Step 5: The autoencoder and two discriminative network modules play concurrently against each other to obtain their objectives.

For example, in order to facilitate dimensionality reduction in scRNA-seq analysis, Lin et al. [27] proposed the DR-A (Dimensionality Reduction with Adversarial variational autoencoder) model (Figure 4), which is a deep learning GAN architecture. Briefly, the DR-A model incorporates a deep adversarial variational autoencoder-based approach, which consists of two deep learning algorithms including the adversarial autoencoder structure [34] and the variational autoencoder structure [14]. More precisely, the DR-A model is a deep adversarial variational autoencoder structure with dual matching, where an additional discriminative network module is designed to single out real scRNA-seq data from the reconstructed scRNA-seq data (Figure 4). In order to overcome the training instability problem, the DR-A model is equipped with the Bhattacharyya distance metric to assess the similarity between probabilities. Additionally, the DR-A model uses a zero-inflated negative binomial (ZINB) distribution structure [81,82], which is well-suited for gene expression data. From the experiments, it was indicated that the DR-A model had better performance than other widely used dimensionality reduction methods, including the PCA [80], Zero-Inflated Factor Analysis (ZIFA) [83], Single-cell Variational Inference (scVI) [82], Sparse Autoencoder for Unsupervised Clustering, Imputation, and Embedding (SAUCIE) [84], T-distributed stochastic neighbor embedding (t-SNE) [85], and Uniform Manifold Approximation and Projection (UMAP) [86]. It should be noted that the scVI and SAUCIE methods also employ deep learning approaches such as deep autoencoder algorithms, but not the GAN architecture. Moreover, the scVI method is based on the variational autoencoder structure [14] and conditional ZINB distributions [87]. On the other hand, PCA, ZIFA, t-SNE, and UMAP utilize traditional machine learning approaches without deep learning techniques, where deep learning techniques usually refer to artificial neural networks with multiple layers, that is, fully connected deep neural network algorithms.

**Figure 5:**
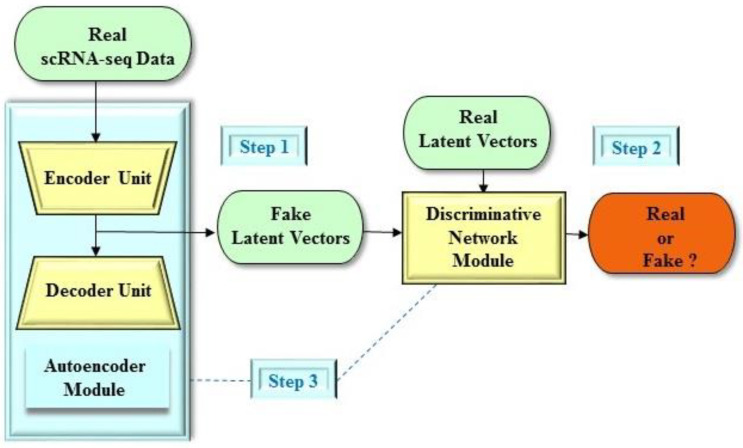
An example of the deep adversarial variational autoencoder structure for dimensionality reduction in scRNA-seq analysis. Step 1: The encoder unit produces synthetic latent vectors as real as possible. The encoder unit provides the mean and covariance of the Gaussian distribution to serve as the variational distribution, which is commonly generated by a variational autoencoder structure. Step 2: The discriminative network module assesses the probability that the latent vector stems from the real latent vectors. Step 3: Both the autoencoder and discriminative network modules play concurrently against each other to obtain their objectives.

In short, the deep adversarial variational autoencoder structure adopts the features of the adversarial autoencoder [34] and variational autoencoder [14] structures (as discussed in the previous section). In the deep adversarial variational autoencoder structure, an autoencoder module consists of a deep encoder unit and a deep decoder unit (Figure 5). In addition, the objective of the deep encoder unit is to generate the mean and covariance of the Gaussian distribution to serve as the variational distribution, which is also normally provided by a variational autoencoder structure [14]. Moreover, the autoencoder module continuously learns to minimize the restoration error and then regenerates the input of the scRNA-seq data to be as credible as possible. It is worthwhile to mention that the deep encoder unit of the adversarial variational autoencoder structure is also considered as the generative network module of the GAN framework. Furthermore, the encoder unit is constructed to fool the discriminative network module of the GAN framework to realize that the latent vector is generated from the true prior distribution. On the contrary, the discriminative network module is concurrently trained to distinguish between the true latent vector and the latent vector generated by the encoder unit (that is, the generative network module). All in all, the deep adversarial variational autoencoder structure is ultimately able to reconstruct the portrayal of the probability distribution of the scRNA-seq data.

Likewise, in order to perform the dimensionality reduction task in scRNA-seq analysis, Ghahramani et al. [88] proposed to use the GAN architecture [32,73,89], which is comprised of a generative network module and a discriminative network module. Essentially, the generative network module is constructed to generate realistic output data based on a randomly-produced latent vector. That is, the objective of the generative network module is to provide a transformation from a lower dimensional space to the higher dimensional space representing the gene expression data. On the contrary, the discriminative network module is arranged to differentiate between the ground true data and the information (that is, the generated latent vector) of the generative network module, simultaneously. Altogether, the goal of the training in the GAN architecture is to make sure that the latent vector, which is generated by the generative network module, matches with specific prior latent distribution.

## 5. *De Novo* Peptide and Protein Design

In this section, we focus specifically on the problem of *de novo* peptide and protein design in drug design and discovery using GAN-based approaches. The goal of *de novo* peptide and protein design is to generate new peptides and proteins based on the physical principles of protein folding [90]. While proteins are macromolecules consisting of sequences of amino acids, a peptide is defined as a short chain of amino acids [90]. While this review does not intend to report all studies in an exhaustive way, it still is representative of the current trend for research in *de novo* peptide and protein design using GAN-based approaches.

There are several studies for *de novo* peptide and protein design in drug design and discovery using GAN-based approaches, including the LSTM-GAN (Long Short-Term Memory Generative Adversarial Network) structure in peptide design [91], the gcWGAN (Guided Conditional Wasserstein Generative Adversarial Network) structure in peptide folding [92], the DCGAN (Deep Convolutional Generative Adversarial Network) structure in protein backbone design [93], the DCGAN structure in target-specific compounds for cannabinoid receptors [94], the GANDALF (Generative Adversarial Network Drug-tArget Ligand Fructifier) structure in peptide design [95], and the Feedback-GAN structure in antimicrobial peptides [96].

For example, Sabban and Markovsky [91] suggested that the LSTM-GAN structure, a combination method involving the GAN architecture and long short-term memory units, was able to generate novel helical protein backbone topologies with preferred features in the context of *de novo* protein design. The LSTM-GAN structure employed a long short-term memory unit in the generative network module and another one in the discriminative network module, where the long short-term memory unit is often utilized in the field of natural language processing [97]. The LSTM-GAN structure was implemented by using the SenseGen software framework, which was originally designed to synthesize sensory data in the fields of data privacy and big data analytics.

To generate novel protein folds with high yields, Karimi et al. [92] also investigated the gcWGAN structure by leveraging the conditional Wasserstein GAN structure, which represents a variant of the GAN-based frameworks. The concept of the conditional Wasserstein GAN structure stems from the Wasserstein GAN structure with gradient penalty [32,73]. The gcWGAN structure consists of the conditional Wasserstein GAN structure and an oracle. In the gcWGAN structure, the generative network module generates sequences, and each sequence receives a predicted fold from the oracle. Then, the predicted folds serve as the feedback to the generative network module.

In addition, a study by Anand and Huang [93] implicated that the DCGAN structure can be applied to generate novel protein structures, which was represented by using a pairwise distance matrix between the alpha-carbons. Here, both the generative and discriminative network modules were implemented by using the convolutional neural network structure, which was originally employed for object recognition and classification in the field of computer vision. Formerly, the DCGAN structure was proposed to learn a hierarchy of image representations using an unsupervised learning scheme in the field of computer vision [89].

Moreover, the subsequent study by Bian et al. [94] reported that the DCGAN structure was able to generate target-specific compounds for cannabinoid by using well-developed convolutional neural network software frameworks such as the LeNet-5 model [98]. Here, both the generative and discriminative network modules were implemented by using convolutional neural networks. Originally, the LeNet-5 software framework (http://yann.lecun.com/exdb/lenet/) was designed for machine-printed and handwritten character recognition by using the convolutional neural network structure, a popular deep learning method in the field of computer vision.

Likewise, Rossetto and Zhou [95] implemented the GANDALF framework to produce new peptides for drug targets, where a generated peptide was highly similar to the FDA approved drugs. In line with two other studies [93,94], the GANDALF framework also employed the DCGAN structure, where five-layer convolutional neural networks were implemented for both the generative and discriminative network modules.

In another study, Gupta and Zou et al. [96] showed that the Feedback-GAN structure, a combination method involving the GAN architecture and a differentiable neural network analyzer, was able to produce antimicrobial peptides with desired properties. The differentiable neural network analyzer is a prediction algorithm to determine if a gene sequence can encode an antimicrobial peptide. In the Feedback-GAN structure, the differentiable neural network analyzer and the GAN architecture are connected by the feedback-loop training mechanism so that the generative network module can generate valid sequences. At each epoch, the generative network module produces several sequences, and each sequence receives a score from the differentiable neural network analyzer. The highest scoring sequence is then selected as the input for the discriminative network module. While the generative network module is trained to generate fake sequence, the discriminative network module receives both real and fake sequences and differentiates whether its input is real or not.

## 6. Limitations

The discoveries as illustrated in the aforementioned sections should be explained by taking into account various disadvantages of these research studies in the interdisciplinary fields of drug design and discovery, artificial intelligence, machine learning, and deep learning. One major disadvantage of these previous studies is that there were no well-defined conclusions because universal benchmark datasets might be previously unavailable to carefully conduct well-thought-out comparisons between various GAN-based frameworks in the past discoveries [99,100]. Recently, two benchmarking tools for molecular *de novo* design were developed, including GuacaMol [101] and MOSES (Molecular Sets) [102]. Since standardized benchmarks have resulted in accelerated progresses in the field of computer vision, it is believed that the field of molecular *de novo* design can also take advantages of standardized benchmarks [101]. The GuacaMol framework defined a suite of benchmarks for molecular *de novo* design, where the training and testing datasets were derived from the ChEMBL database (https://www.ebi.ac.uk/chembl/) [101]. By using the GuacaMol framework, it was revealed that the adversarial autoencoder structure outperformed ORGAN [36] in terms of five benchmarks such as validity (0.822 vs. 0.379), uniqueness (1.000 vs. 0.841), novelty (0.998 vs. 0.687), Kullback–Liebler divergence (0.886 vs. 0.267), and Fréchet ChemNet Distance (0.529 vs. 0.000) [101]. Fréchet ChemNet Distance evaluates the difference in the distribution of molecules between the generated dataset and the training dataset [103]. On the other hand, the MOSES framework was based on the ZINC database [54] and various benchmarks such as validity, uniqueness, Fréchet ChemNet Distance, fragment similarity, scaffold similarity, and similarity to a nearest neighbor [102]. By utilizing the MOSES framework, it was indicated that the adversarial autoencoder structure had performance metrics such as validity (0.937), uniqueness (0.997), novelty (0.793), and Fréchet ChemNet Distance (0.556). It is obvious that evaluation results from these two benchmarking frameworks are quite different even when using the same model/structure (that is, the adversarial autoencoder structure). Thus, universal benchmark frameworks are warranted for molecular *de novo* design.

One main disadvantage of the GAN architecture is that it is hard to converge and massively unstable when training the GAN architecture [104]. Because the convergence of the GAN architecture may often fail, it requires a relatively large amount of computing and human efforts to achieve successful training [104]. Another key issue is the mode collapse problem in the GAN architecture, where the generative network module is only trained to fool the discriminative network module and is unable to capture the multimodal distributions of the real data [105].

It should be noted that the following limitations may be attributed to not only the GAN architecture but also any generative approaches. As the GAN architecture employs deep neural networks, the common problems in deep neural networks may also be included in the GAN architecture [31]. For instance, the accuracy of the GAN-based structure would be rather low when the sample size is limited [31]. Additionally, it is crucial to generalize to independent datasets for various experiments by employing common benchmark datasets [99,100]. Yet, it is an open challenge that large-scale benchmark datasets [106,107] might not be accessible to facilitate subsequent analysis in deep learning research. Thus, future deep learning research such as GAN-based frameworks should be reliably reproducible based on commonly well-accepted benchmark datasets, which should be carried out by the research community in the interdisciplinary fields of drug design and discovery, artificial intelligence, machine learning, and deep learning.

Yet another pitfall is that it is particularly challenging to figure out the interpretation of deep learning approaches such as the GAN architecture. In general, deep learning algorithms are considered as a “black box”, which is normally difficult to interpret [9]. It is also worth mentioning that not only the GAN architecture but also other deep learning approaches (such as recurrent neural network and convolutional neural network models) carry this concern [9]. It is evident that more interpretable deep learning approaches are warranted, and we could thereby pinpoint explicable features extracted from the GAN architecture.

Furthermore, it should be emphasized that we should employ the traditional linear models as the fundamental basis when applying deep learning approaches such as the GAN architecture [108]. That is, deep learning approaches such as the GAN architecture should be a compatible approach not only to the traditional linear models but also to non-linear models such as support vector machines and random forests.

Moreover, a common pitfall is that the aforementioned GAN-based frameworks may not employ the cross-validation strategy to avoid the risk of overfitting during the training step. For instance, the repeated 10-fold cross-validation method and leave-one-out cross-validation method could be good procedures for examining the generalization of GAN-based frameworks [109,110]. In brief, the repeated 10-fold cross-validation method randomly separates the whole dataset into ten subsets, and then the GAN-based frameworks can be trained by nine-tenths of the data and tested by the remaining tenth of data [111]. Next, the previous step is repeated nine more times by choosing different nine-tenths of the data for training and a different tenth of the data for testing. Similarly, the leave-one-out cross-validation method is an extreme case where the number of folds is equal to the number of samples in the whole dataset [112]. The leave-one-out cross-validation method is usually adopted when the number of samples in the whole dataset or in a particular subset is small [112]. Nonetheless, we hypothesize that the cross-validation strategy may be supposed to influence the long-term structure and performance in the eventual GAN framework [113].

Due to the great range of various molecular representations, comparison of molecular representations are extensively discussed in the literature [114,115,116]. Since the molecular representation contains the high information content per se, it is expected that more simplistic representations will continue to be less informative than the complex ones (even when using the GAN architecture) [114,115,116]. However, this remains to be explored. It should be also pointed out that future research is needed to investigate and compare multiple molecular representations, such as SMILES strings and graph grammar-based approaches, in order to properly represent the chemical structures and further improve chemical accuracy [117].

Of note, nowadays only a handful of applications have been investigated by using the GAN architecture to date in the interdisciplinary fields of drug design and discovery, artificial intelligence, machine learning, and deep learning. Due to our goal in this review, only three main arenas in drug design and discovery using the GAN architecture have been described, including molecular *de novo* design, dimension reduction of single-cell data in preclinical development, and *de novo* peptide and protein design. Although in the present review we only presented several research reports to depict the related GAN-based frameworks in these three applications, it is highly anticipated that the GAN architecture would be applied to other research areas in drug design and discovery such as compound property and activity prediction, reaction analysis, synthesis prediction, and biological image analysis in the near future [1].

There are two major open challenges and emerging problems in the GAN architecture itself. The first open challenge and emerging problem in the GAN architecture is to resolve the mode collapse problem, especially for extremely complicated multimodal distributions in the real data [105]. The second open challenge and emerging problem in the GAN architecture is to overcome the training instability problem, where the convergence of the cost functions in the generative and discriminative network modules is prone to be unstable [104].

One major open challenge and emerging problem in molecular *de novo* design using the GAN architecture is that open-source software frameworks are crucially needed because of importance in reusability and replicability [118]. Secondly, universal benchmarking metrics in molecular *de novo* design using the GAN architecture could be very challenging. It was suggested that the metrics from the GAN-based models such as the Wasserstein distance could be a good candidate to evaluate the molecular compounds generated by the GAN architecture [118]. It should be emphasized that the aforementioned challenges and emerging problems in molecular *de novo* design using the GAN architecture may also stem from any generative models.

It has been suggested that one of the major challenges and emerging problems in drug design and discovery using artificial intelligence and machine learning technologies is the lack of big data in the various phases of research and development for candidate molecular compounds, such as pharmacokinetic and pharmacodynamic analysis [119]. In general, this fact also holds true for the GAN architecture because the meaningful datasets would be required to train various GAN-based frameworks at the first place. Another potential challenge and emerging problem in drug design and discovery using artificial intelligence and machine learning technologies, which also holds true for the GAN architecture, is that overall datasets from failed clinical trials (that is, true negative data) are unavailable for the research community [119].

## 7. Other Relevant Applications in Drug Design and Discovery

In the previous sections, we mention a wide variety of research studies in drug design and discovery using various GAN-based frameworks including molecular *de novo* design, dimension reduction of single-cell data in preclinical development, and *de novo* peptide and protein design. While this review does not intend to cover all applications that have been studied in an exhaustive manner, it nevertheless is representative of the general trend for current research in drug design and discovery using GAN-based approaches. However, it is arguable that what applications could likely be considered as the focus of attention in the field of drug design and discovery nowadays.

For other relevant research studies in drug design and discovery, the reader can refer to a recent review by Chen et al. [1] for various applications using other deep learning approaches. The reader can also refer to a recent review by Hessler and Baringhaus [3] for other aspects of drug design in artificial intelligence and machine learning technologies, such as compound property prediction and synthesis prediction, which again are not the focus of this review.

For example, Chen et al. [1] indicated that other feasible deep learning approaches include convolutional neural network algorithms [62], recurrent neural network algorithms [46], and fully connected deep neural network algorithms [120]. In addition, these three deep learning approaches could be applied to other research categories in drug design and discovery, including compound property and activity prediction, reaction analysis, synthesis prediction, and biological image analysis [1,121,122].

On another note, in drug design and discovery, a new candidate molecule must be tested through various criteria such as physicochemistry, absorption, distribution, metabolism, excretion, and toxicity properties [3]. Numerous traditional machine learning models such as random forests and support vector machines have shown to perform well in property prediction (such as biological activity prediction and physicochemical parameters) [3]. However, Hessler and Baringhaus [3] suggested that deep learning models such as fully connected deep neural network algorithms outperformed traditional machine learning methods for property prediction. For instance, Ma et al. [123] employed fully connected deep neural network algorithms for property prediction by using the Kaggle benchmark datasets (www.kaggle.com/datasets). It has been shown that fully connected deep neural network algorithms had a 10% improvement in terms of the squared Pearson correlation coefficient when compared with random forests [123].

Furthermore, based on benchmark results, Chen et al. [1] implicated that the multi-task deep neural network model exceled the single-task deep neural network model and traditional machine learning models in compound property prediction, where these models were tested by using the drug-like compound datasets from the ChEMBL database. For example, to overcome the issues of small and/or noisy training datasets, a multi-task deep neural network framework called TopologyNet [124] was proposed for biomolecular property prediction by using the intrinsic relatedness and predictive information among multiple prediction tasks. It has been demonstrated that under the condition of relatively small training datasets, TopologyNet (Pearson correlation coefficient of 0.826) was able to achieve better performance than single-task models such as random forests (Pearson correlation coefficient of 0.803) [124].

## 8. Conclusions and Perspectives

As indicated by the aforementioned findings, the GAN architecture affirms to provide novel approaches for drug design and discovery including molecular *de novo* design and dimension reduction of single-cell data in preclinical development. Firstly, in terms of molecular *de novo* design techniques, it is of great interest that future prospective research projects should concern deep learning approaches such as the GAN architecture to generate novel molecular compounds with desired molecular features, which may contribute to feasible medical solutions in public health as well as global health. Secondly, in terms of dimension reduction of single-cell data, it is also important to note that the deep GAN architecture may play a key role in the pivotal stage prior to suitable downstream analysis of scRNA-seq data during the pipeline of preclinical drug development. Furthermore, deep learning approaches such as the GAN architecture will be assuredly created towards the field of drug design and discovery in light of the pressing needs of innovative techniques in the fields of global health, public health, and population health [125]. Thereby, we would expect that the recent advancements in single cell sequencing technologies and data-intensive health sciences might undoubtedly trigger novel deep learning software frameworks, such as the GAN architecture, for global health, public health, and population health over the next few years [126,127]. Thus, the general public and governments should deal with these challenges and issues with high priorities in the up-coming decade [128,129]. In the next generation to come, the drug design and discovery pipeline involving deep learning approaches such as the GAN architecture would become a reality in drug-specific clinical care when prospective large-scale studies are able to comprehensively evaluate the relevant novel molecular compounds as well as single-cell attributes [130,131].

## Figures and Tables

**Table 1:** Relevant studies on the GAN-based structures of molecular *de novo* design.

Study	Structure	Architecture	Object Generated	Learning Technique	Databases	Results
Kadurin et al. [28,29]	druGAN	AAE	latent vector	autoencoder	PubChem	druGAN generated novel molecular compounds which can be considered as potential anticancer agents.
Guimaraes et al. [36]	ORGAN	GAN	SMILES	RL	ZINC, GDB-17	ORGAN performed better than recurrent neural networks or GAN alone.
Sanchez-Lengeling et al. [37]	ORGANIC	GAN	SMILES	RL	ZINC, GDB-17	ORGANIC showed good performance in terms of the quantitative estimate of drug-likeness, but not the Lipinski’s Rule-of-Five.
Putin et al. [38]	RANC	GAN	SMILES	RL	ZINC, ChemDiv	RANC was superior to ORGANIC in terms of several drug discovery metrics.
Putin et al. [39]	ATNC	GAN	SMILES	RL	ChemDiv	ATNC performed better than ORGANIC in terms of various functions.
Polykovskiy et al. [40]	ECAAE	AAE	latent vector	autoencoder	ZINC	ECAAE generated novel molecular compounds which can be considered as target drugs in rheumatoid arthritis, psoriasis, and vitiligo.
Cao and Kipf [41]	MolGAN	GAN	graph	RL	QM9	MolGAN outperformed ORGAN and variational autoencoder-based structures.
Guarino et al. [42]	DiPol-GAN	GAN	graph	RL	QM9	DiPol-GAN had 1.3 times higher drug-likeliness scores than MolGAN.
Prykhodko et al. [43]	LatentGAN	GAN	SMILES	autoencoder	ChEMBL	LatentGAN created novel drug-like compounds and was compatible to recurrent neural networks.
Maziarka et al. [44]	Mol-CycleGAN	GAN	latent vector	direct flow	ZINC, ChEMBL	Mol-CycleGAN outperformed the junction tree variational autoencoder and the graph convolutional policy network structures.
Méndez-Lucio et al. [45]	Conditioned GAN	GAN	latent vector	direct flow	L1000	Conditioned GAN produced molecular compounds with desired gene expression signatures.

AAE = adversarial autoencoder; ATNC = Adversarial Threshold Neural Computer; druGAN = drug Generative Adversarial Network; ECAAE = Entangled Conditional Adversarial AutoEncoder; GAN = Generative Adversarial Network; LatentGAN = Latent Generative Adversarial Networks; MolGAN = Molecular Generative Adversarial Network; Mol-CycleGAN = Molecular Cycle Generative Adversarial Network; ORGAN = Objective-Reinforced Generative Adversarial Networks; ORGANIC = Objective-Reinforced Generative Adversarial Network for Inverse-design Chemistry; RANC = Reinforced Adversarial Neural Computer.
